# Variations of Training Workload in Micro- and Meso-Cycles Based on Position in Elite Young Soccer Players: A Competition Season Study

**DOI:** 10.3389/fphys.2021.668145

**Published:** 2021-04-29

**Authors:** Hadi Nobari, Reyhaneh Vahabidelshad, Jorge Pérez-Gómez, Luca Paolo Ardigò

**Affiliations:** ^1^Department of Physical Education and Sports, University of Granada, Granada, Spain; ^2^HEME Research Group, Faculty of Sport Sciences, University of Extremadura, Cáceres, Spain; ^3^Department of Exercise Physiology, Faculty of Sport Sciences, University of Isfahan, Isfahan, Iran; ^4^Sports Scientist, Sepahan Football Club, Isfahan, Iran; ^5^Faculty of Motor Sciences, Université libre de Bruxelles, Brussels, Belgium; ^6^School of Exercise and Sport Science, Department of Neurosciences, Biomedicine and Movement Sciences, University of Verona, Verona, Italy

**Keywords:** playing position, training load, monitoring, performance, soccer

## Abstract

The objectives of this study were to quantify the matches and training workload in micro-cycles of an elite young soccer team considering field position and to explain meso-cycles based on change of weekly acute (wAWL), chronic load (wCWL), acute-to-chronic workload ratio, training monotony (wTM), and training strain (wTS) between early-, mid-, and end-season periods considering playing position and whole team. Twenty-six under-16 elite young soccer players participated in this study, including six wide defenders and wide midfielders (WM), five central defenders (CD) and central midfielders, and four strikers (ST). Daily monitoring was performed by players for 20 weeks with the rating of perceived exertion using the Borg CR-10 scale. In comparison with early-season, results showed that there was a significant increase, in all playing positions, in wAWL and wCWL (except ST) and in wTM (except CD and ST) compared with end-season. On the other hand, there were significant reductions in wTS in CD, WM, and ST at the end-season. According to the results, coaches should consider the field position in different situations. Differences between training workload and matches can be a good guide for coaches, who have a special understanding of what causes the most load in training programs. Excessive training workload can potentially cause injury to adolescent athletes and controlling wTM can prevent this.

## Introduction

Training loads (TL) can be categorized internally and externally; the external training load is the specific training prescribed by the micro-electromechanical systems (e.g., global positioning system, location position system, inertial measurement unit), whereas the internal TL is the individual’s physical, mental, and psychological response (Charlot et al., 2016). The monitoring of TL provides valuable information to ensure players are recovering effectively, and also helps to comprehend how a player adapts to, and reacts to, training. Individualization is rarely seen in soccer as training prescriptions are often based on the group (Weston, 2018). Furthermore, the ability to effectively communicate data on TL is very important—data should be analyzed and can be effectively interpreted into clear, practical messages (Clemente et al., 2017).

Exercise volume in sports, such as soccer, is defined as the total duration of minutes or the number of repetitions of exercises (McGuigan and Foster, 2004). The second component of exercise load is usually described using heart rate, lactate concentration measurement, or rating of perceived exertion (RPE) (Foster, 1998; Eniseler, 2005; Dellal et al., 2008, 2010). Furthermore, another way to quantity the level of fatigue, stress, delayed onset muscle soreness, and the quality of sleep is the Hooper Index (Hooper and Mackinnon, 1995; Nobari et al., 2020a, 2021b). Most professional teams are often reluctant to share supervisory data for competitive gain and, as a result, long-term, more detailed approaches are essentially needed to develop specific expertise on how to manage the burden on elite youth soccer (Kelly et al., 2020).

Regular and continuous monitoring of TL allows individuals to control the training response to the load and this helps coaches to analyze the athlete’s fitness daily changes over training and competitions (Gabbett, 2020; Nobari et al., 2020e). In addition, you may want to know if the dose-response is appropriate to allow coaches to keep athletes away from risk of exposure or overexposure to stress (Foster, 1998).

Depending on the level of the competition and team, soccer players should be prepared to carry out three to seven training sessions (Rico-González et al., 2020) and one match once a week (Morgans et al., 2014b). So, the monitoring of internal and external TL helps coaches to design an effective individual and group training periodization in elite team sports (Djaoui et al., 2017; Stevens et al., 2017; Nobari et al., 2020d). The weekly load planning requires attention and scientific support to warrant peak performance in the official match and also to prevent enhanced levels of fatigue and higher risk of injury (Jones et al., 2017; Nobari et al., 2020b).

A workload index can then be calculated indicating whether the individual’s weekly acute workloads (wAWL) is greater than, less than, or equal to the preceding weekly chronic workload (wCWL) the individual has been prepared for Hulin et al. (2016) and Nobari et al. (2020e). Such an index is represented by the acute-to-chronic workload ratio (wACWLR), which is an index of athletes’ training stress, that evolves in relation to the fitness level in response to a training session as accrued through their chronic exposure to training (Gabbe et al., 2006). The wACWLR was designed to help practitioners to better manage athletes’ readiness for competitions, taking into account the risk of non-functional over-reaching and injury (Hulin et al., 2014; Nobari et al., 2020b, 2021c).

In particular, considering low and high wCWL, the player with high wCWL has more resistance to injury during competition and training in a variety of team sports. For this purpose, session RPE (s-RPE) and external measures (viz., tracking variables collected with, i.e., global satellite navigation systems, inertial measurement units, and local positioning systems) are used (Myers et al., 2019; Enright et al., 2020; Griffin et al., 2020). Nowadays, researchers study the data collected during short training micro-cycles of 1 to 3 weeks (Impellizzeri et al., 2005; Morgans et al., 2014a; Anderson et al., 2016; Clemente et al., 2017), meso-cycles of 4 to 10 weeks (Gaudino et al., 2013; Scott et al., 2013), and over longer training periods of 3–4 months (Alexiou and Coutts, 2008; Casamichana et al., 2013) and 10 months (Morgans et al., 2014a).

Although Nobari et al. (2020b, c) and Djaoui et al. (2017) studied the above-mentioned workloads variables, including weekly training monotony (wTM) and training strain (wTS), there is still a paucity of these data available regarding elite soccer players. Therefore, the current study aimed at investigating the variations of training workload in micro- and meso-cycles, based on position, in elite young soccer players. Three objectives were defined for this study: (1) to describe daily pattern and comparisons between every weekday and match day (MD) of the TL status during a common competition micro-cycle for the whole team, considering playing position; (2) to analyze the differences of wAWL, wCWL, wACWLR, wTM, and wTS between meso-cycles (early−, mid−, and end-season periods) considering playing position; and (3) to compare the training workload variables during competition meso-cycles considering the whole team.

## Materials and Methods

### Participants

In this study, twenty-six young soccer players took part (mean ± standard deviation [SD], chronological age 15.5 ± 0.2 years, height 172.9 ± 4.2 cm, body mass 61.4 ± 5.6 kg, body fat 8.6 ± 2.9% maximum oxygen uptake (VO_2__max_) 48.4 ± 2.4 ml⋅kg^–1^⋅min^–1^, maturity offset 1.9 ± 0.3 years and peak height velocity age (PHV) 13.6 ± 0.3 years). Players participated in the national competitions of Iran under 16 s competitions. For analyzing the differences between player positions, it was differentiated between six wide defenders (WD) and wide midfielders (WM), five central defenders (CD) and central midfielders (CM), and four strikers (ST; Malone et al., 2015; Nobari et al., 2020e). Their characteristics are displayed in Table 1. In this study, inclusion criteria were: (i) players who have attended at least 90% of the training seasons; (ii) players who did not participate in another training program; and (iii) each player, who did not participate in the match for a week, had to practice in a separate session without the ball or in small-sided games. The Ethics Committee of the University of Isfahan and University of Mohaghegh Ardabili approved this study. Both players and their parents completed the consent form and signed it in accordance with the Helsinki Declaration.

**TABLE 1:** Absolute size anthropometric, body composition, and maturation of soccer player by playing positions. Mean ± standard deviation (SD).

Characteristics	field position									
	WD = 6N		CD = 5N		CM = 5N		WM = 6N		ST = 4N	
	mean	SD	mean	SD	mean	SD	mean	SD	mean	SD
Anthropometric										
Age (years)	15.4	0.3	15.5	0.3	15.4	0.2	15.4	0.3	15.6	0.1
Height (cm)	175.8	4.0	174.4	5.0	169.8	1.3	171.2	2.9	172.8	5.3
Body mass (kg)	66.9	5.7	62.6	4.9	58.1	1.4	57.5	3.9	61.4	6.1
BMI (kg⋅m^–2^)	22.2	1.5	21.0	1.2	20.1	0.4	19.3	1.2	20.8	1.9
VO_2__*max*_ (ml⋅kg^–1^⋅min^–1^)	48.0	3.2	48.1	1.3	47.6	1.8	49.4	2.7	49.1	2.9
Career (years)	6.0	1.5	7.4	1.1	6.0	1.4	5.3	1.4	6.5	1.9
Maturations (years)										
Maturity offset	2.1	0.2	1.8	0.4	1.8	0.1	1.7	0.4	1.9	0.2
PHV	13.4	0.4	13.6	0.4	13.6	0.2	13.7	0.4	13.6	0.3
Body compositions										
BF%	8.2	2.2	10.3	3.8	10.4	3.3	6.1	1.9	8.6	0.6
BF (kg)	5.6	1.8	6.4	2.4	6.0	1.9	3.5	1.1	5.3	0.8
LBM (kg)	61.4	4.1	56.2	5.6	52.1	2.3	53.9	3.9	56.1	5.3

*N, number of players; WD, wide defenders; WM, wide midfielders; CD, central defenders; CM, central midfielders; ST, strikers; BMI, body mass index; VO_2max_, maximal oxygen consumption; PHV, peak height velocity age; BF, body fat; LBM, lean body mass.*

### Sample Size

The power and sample for a Fischer test was estimated. According to the statistically analyzed method, it resulted in a within-group factor in repeated measurements amounting to 98.1% (real power) probability of correct rejection of the null hypothesis, with no difference in the results of monitoring the workload of training over time with 25 players.

### Experimental Approach to the Problem

This is a descriptive-longitudinal study regarding the full match monitoring season of a soccer team. Daily monitoring was observed regarding the players from the beginning of the match season for 20 weeks. The whole season was divided into three meso-cycles according to the starting team competition schedule: (1) early-season, week (W) 1 to W7; (2) mid-season, W8 to W13; and (3) end-season, W14 to W20. This was done to analyze the differences between meso-cycles with and without considering playing position. Description of the typical micro-cycle pattern and related analysis, only according to the data of those weeks of the competition, was done. This was done regarding the repeated training pattern and including only one match per week. Players had experience using the RPE, at least, over the two previous years. They individually reported the RPE score 30 min after training and competition. Then, TL was calculated considering s-RPE and training time for each training session. These data were used to obtain information and analyze weekly workload parameters (wAWL, wCWL, wACWLR, wTM, and wTS; Malone et al., 2015; Nobari et al., 2020e). The wCWL and wACWLR were calculated with the uncoupled formula after the third and fourth weeks, respectively. For calculating the VO_2__max_ of the players, the 30–15 Intermittent Fitness Test (30–15IFT; Buchheit, 2010) was performed in the pre-season.

### Measurements

#### Anthropometry and Body Composition

All anthropometric measurements, body composition, calculation of body fat percentage, and how to calculate maturity offset and age at peak velocity have been performed based on the methods of previous studies (Nobari et al., 2020e, 2021a,c,d). All measurements were performed by an expert with 5 years of experience in the field (Arazi et al., 2015; Rahmat et al., 2016).

### Aerobic Power Test

30–15 Intermittent Fitness Test was administered to estimate the VO_2__max_ and thus fitness level of the individuals. Such a test includes a 40-meter shuttle to be covered in 30 s followed by 15 s of passive recovery. The first step therefore lasts 30 s with the first speed amounting thus to 8 km⋅h^–1^ and then increasing by 0.5 km⋅h^–1^ every 45 s (Buchheit, 2010). To warm up before all the tests, players performed standard warm-ups for 10 min including jogging, dynamic stretching, some ABC run drills, and short submaximal-speed runs under surveillance of the fitness coach of the team. After warming up, the subjects in groups of four stood before line A. After two speakers played “Get ready, go!,” they started running back and forth between lines B and C for 30 s. Then, the subjects moved on to line A for the next stage. The test continued until the subjects could continue or were not able to cope with the imposed speed for three consecutive shuttles. The 30-15IFT was used to estimate the VO_2__max_ with the following formula (Buchheit, 2010): VO_2__max_ (ml⋅kg^–1^⋅min^–1^) = 28.3−(2.15 × 1)−(0.741 × 16 years)−(0.0357 × mass) + (0.0586 × 16 years × VIFT) + (1.03 × VIFT), where VIFT is the final running speed.

#### Monitoring Internal Training Workloads

Prior to the research, players were introduced to the RPE scale. Players were monitored daily in terms of RPE using the Borg CR-10 scale (Nobari et al., 2020e). The validity and reliability of this scale have been demonstrated in a previous study to estimate s-RPE (Malone et al., 2015). The question was “How intense was your session?.” The players responded to this question 30 min after the end of training or match session (Foster et al., 2021). Durations of training sessions were measured. S-RPE was calculated by multiplying the CR-10 score, on a scale of 10, by the duration of the session in min and was used as a measure of internal load. Data collection was done with each player separately, to avoid other players hearing score points. The data record was made daily in Excel.

#### Training Workload Calculation

In this study, workload variables were calculated as follows: (i) wAWL, considering all daily TL each week; (ii) wACWLR, the uncoupled formula to calculate this variable was used to decrease the reporting error (for example, such a formula was used to calculate the wACWLR4 = wAWL 4/0.333 × (wAWL 2 + 3 + 4); (iii) wTM, to calculate this variable the following calculation was operated: wAWL was divided by the SD of same wAWL); and (iv) wTS, to calculate this variable, the following calculation was operated: wAWL was multiplied by wTM of the same week.

### Statistical Analyses

Descriptive statistics were presented as mean ± SD. Shapiro-Wilk and Levene’s tests were performed to check the normality and consistency of the data, respectively. Variations between the three meso-cycle were evaluated using a repeated-measures analysis of variance (ANOVA), followed by Bonferroni *post hoc* test for pairwise comparisons [(group × period) to compare (group) and (group × period) and to compare (period)]. Partial eta squared (η*p*^2^) was calculated as effect size regarding the repeated-measure ANOVA. Similar methods were used to analyze the possible differences between every weekday and the MD in terms of TL during a common micro-cycle of competition [(group × day) to compare (group) and (group × day) and to compare (day)]. Hedge’s *g* effect size with 95% confidence interval was also calculated to determine the amount of pairwise comparisons between meso-cycles. The Hopkins threshold was utilized as follows: *g* ≤ 0.2, trivial; 0.2 < *g* ≤ 0.6, small; 0.6 < *g* ≤ 1.2, moderate; 1.2 < *g* ≤ 2.0, large; 2.0 < *g* ≤ 4.0, very large; and *g* > 4.0, nearly perfect (Hopkins et al., 2009). Significance level was set at *p* ≤ 0.05. The Statistical Package for Social Sciences (SPSS version 25.0, IBM SPSS Inc., Chicago, IL, United States) was used for calculations. It was calculated on an *a priori* estimation of power and sample size. For this purpose, the statistical software G-Power (University of Dusseldorf, Dusseldorf, Germany) was used. The selected study plan was therefore: *F*-test regarding ANOVA for repeated measures and within factors, power α of error probability of 0.05, power 1-β of error probability of 0.95, number of groups and measurements five and three, respectively.

## Results

**FIGURE 1:**
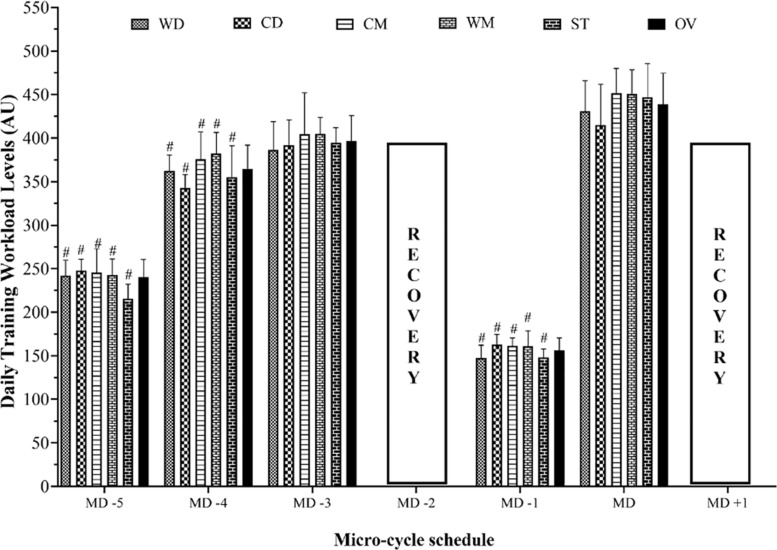
Micro-cycle pattern and comparisons over each day of the training workload during a competition season considering field position and whole team. # Significant differences with *p* ≤ 0.05 between MD and training days. WD, wide defenders; WM, wide midfielders; CD, central defenders; CM, central midfielders; ST, strikers; OV, whole team; MD, match day.

Figure 1 displays the micro-cycles and comparisons between every training day and the MD in terms of TL during a main competition cycle considering playing position. The results of repeated-measures ANOVA were performed with two models. Analysis of differences between TL between players’ positions was performed on a daily basis. No differences were found in the comparisons within weekdays between the playing positions. Analyzing the differences between the days, a significant difference between the TL on MD_–__5_, MD_–__4_ and MD_–__1_ (5, 4, and 1 days before match day, respectively; *p* ≤ 0.001) compared with MD regarding all playing positions was observed. There was no significant difference between the MD compared with MD_–__3_.

**FIGURE 2:**
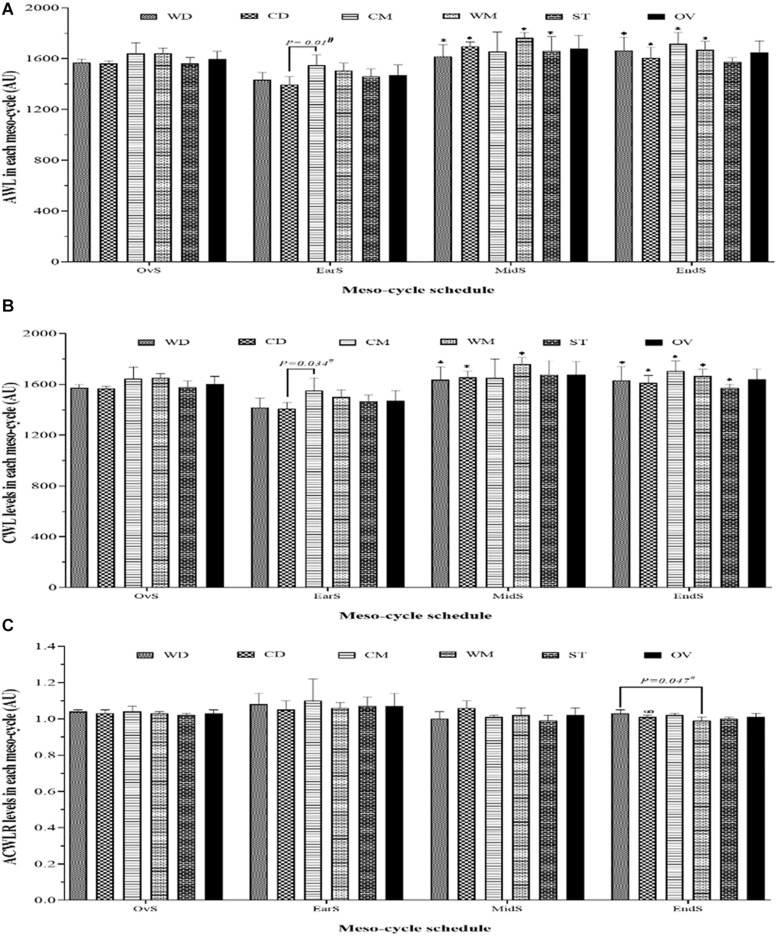
Weekly acute **(A)** and chronic workload **(B)** and weekly acute-to-chronic workload ratio **(C)** meso-cycle patterns and comparisons over each period during a competition season considering field position and whole team. * represents a statistically significant difference comparing with Ear-S (*p* ≤ 0.05); ∞ represents a statistically significant difference comparing with Mid-S (*p* ≤ 0.05); # significant differences between two field positions in the same period of the season (*p* ≤ 0.05). AU, arbitrary units; WD, wide defenders; WM, wide midfielders; CD, central defenders; CM, central midfielders; ST, strikers; OV, whole team; EarS, early-season; MidS, mid-season; EndS, end-season; AWL, weekly acute workload; CWL, weekly chronic workload; ACWLR, weekly acute-to-chronic workload ratio.

Figure 2 illustrates the wAWL, wCWL, and wACWLR variations of the different meso-cycles considering playing position. The highest and the lowest recorded values occurred regarding wAWL in mid-season [WM, 1764.8 ± 8.4 arbitrary units (A.U)] and early-season (CD, 1394.9 ± 63.2 A.U), regarding wCWL in mid-season (WM, 1759.2 ± 50.4 A.U) and early-season (CD, 1405.9 ± 51.0 A.U), and regarding wACWLR in early-season (CM, 1.10 ± 0.12 A.U) and mid-season (ST = 0.99 ± 0.03 A.U). There were significant wAWL and wCWL decreases in all playing position, except ST, comparing early- with end-season. Furthermore, there was a significant wACWLR decrease from mid- to end-season only in CD (*p* = 0.041) playing position.

**FIGURE 3:**
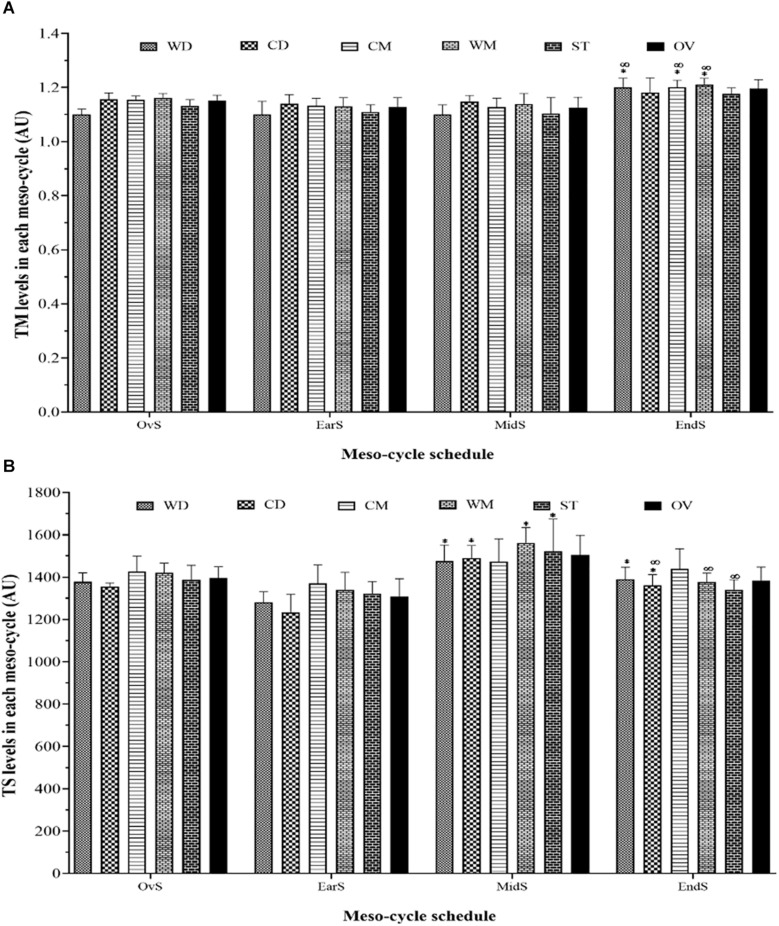
Weekly training monotony **(A)** and training strain **(B)** meso-cycle patterns and comparisons over each period during a competition season considering field position and whole team. * represents a statistically significant difference comparing with Ear-S (*p* ≤ 0.05); ∞ represents a statistically significant difference comparing with Mid-S (*p* ≤ 0.05). AU, arbitrary units; WD, wide defenders; WM, wide midfielders; CD, central defenders; CM, central midfielders; ST, strikers; OV, whole team; EarS, early-season; MidS, mid-season; EndS, end-season; TM, weekly training monotony; TS, weekly training strain.

The comparisons between the different playing positions are displayed in Figure 3 considering wTM and wTS over each meso-cycle of the competition season. The highest and the lowest recorded values occurred regarding wTM in end-season (WM, 1.21 ± 0.02 A.U) and mid-season (WD, 1.10 ± 0.03 A.U) and regarding wTS in mid-season (WM, 1562.5 ± 72.8 A.U) and early-season (CD = 1233.0 ± 86.1 A.U). Overall, results revealed that there was a significant wTM increase comparing early- with end-season for all playing positions except CD and ST. On the other hand, there was a significant wTS decrease comparing mid-season with end-season in CD, WM, and ST.

Results of repeated-measure ANOVA revealed differences over competition season meso-cycles in terms of wAWL (*p* < 0.001, η*p*^2^ = 0.598), wCWL (*p* < 0.001, η*p*^2^ = 0.602), wACWLR (*p* < 0.001, η*p*^2^ = 0.366), wTM (*p* < 0.001, η*p*^2^ = 0.495), and wTS (*p* < 0.001, η*p*^2^ = 0.601). Table 2 presents the pairwise comparisons between all in-season periods in terms of wAWL, wCWL, wACWLR, wTM, and wTS. Overall, a significant increase in all variables was observed over season meso-cycles. This highlights the importance of reducing the variables from mid-season to the end of the season. Only three comparisons did not present any differences and namely no significant difference was observed in terms of wAWL and wACWLR from mid- to end-season and in terms of wTM from early- to mid-season (*p* > 0.05).

**TABLE 2:** Comparisons over competition season meso-cycles in terms of training workload variables. Mean ± standard deviation.

Variables	Season period		Comparison	Mean difference (95% CI)	*p*	Hedge’s *g* (95% CI)
**Wawl** (AU)	Ear-S	1469.3 ± 79.9	Ear-S *vs* Mid-S	210.8 [159.4 to 262.3]	**< *0.001***	−0.3 [−0.8 to 0.3]
	Mid-S	1680.2 ± 103.3	Ear-S *vs* End-S	181.8 [134.7 to 228.8]	**< *0.001***	2.0[1.3 to 2.6]
	End-S	1651.1 ± 88.9	Mid-S *vs* End-S	−29.1 [−82.8 to 24.6]	*0.436*	−0.3 [−0.8 to 0.3]
**wCWL** (AU)	Ear-S	1467.5 ± 83.4	Ear-S *vs* Mid-S	208.6 [156.5 to 260.8]	**< *0.001***	2.1[1.4 to 2.7]
	Mid-S	1676.2 ± 102.8	Ear-S *vs* End-S	172.0 [126.3 to 217.7]	**< *0.001***	1.9[1.3 to 2.6]
	End-S	1639.6 ± 80.6	Mid-S *vs* End-S	−36.6 [−88.1 to 14.8]	**< *0.001***	−0.4 [−0.9 to 0.2]
**wACWL**R (AU)	Ear-S	1.07 ± 0.07	Ear-S *vs* Mid-S	−0.05 [−0.08 to −0.02]	**< *0.001***	−0.9 [−1.5 to −0.3]
	Mid-S	1.02 ± 0.04	Ear-S *vs* End-S	−0.06 [−0.09 to −0.03]	**< *0.001***	−1.1 [−1.7 to −0.6]
	End-S	1.01 ± 0.02	Mid-S *vs* End-S	−0.01 [−0.03 to 0.01]	*0.433*	−0.2 [−0.8 to 0.3]
**wTM** (AU)	Ear-S	1.13 ± 0.03	Ear-S *vs* Mid-S	−0.004 [−0.02 to 0.02]	< *0.999*	−0.1 [−0.6 to 0.4]
	Mid-S	1.12 ± 0.04	Ear-S *vs* End-S	0.07 [0.05 to 0.09]	**< *0.001***	1.8[1.2 to 2.5]
	End-S	1.20 ± 0.03	Mid-S *vs* End-S	0.07 [0.05 to 0.09]	**< *0.001***	1.8[1.1 to 2.4]
**wTS** (AU)	Ear-S	1309.4 ± 83.6	Ear-S *vs* Mid-S	195.8 [146.5 to 245.2]	**< *0.001***	2.0[1.4 to 2.7]
	Mid-S	1505.2 ± 93.3	Ear-S *vs* End-S	74.5 [33.0 to 115.9]	**< *0.001***	0.9[0.4 to 1.5]
	End-S	1383.8 ± 64.1	Mid-S *vs* End-S	−121.4 [−166.0 to 076.8]	**< *0.001***	−1.4 [−2.0 to −0.8]

*AU, arbitrary units; CI, confidence interval; wAWL, weekly average acute workload in AU; wCWL, weekly average chronic workload in AU; wACWLR, weekly average acute:chronic workload ratio; wTM, weekly average training monotony in AU; wTS, weekly average training strain in AU; Ear-S, early-season period; Mid-S, mid-season period; End-S, end-season period; P, *p*-value at alpha level 0.05; Hedges’s *g* (95% CI), Hedges’s *g* effect size magnitude with 95% confidence interval. Significant differences (*p* ≤ 0.05) are highlighted in bold.*

## Discussion

A primary objective of the present study was to investigate the wAWL, wCWL, wACWLR, and wTM and wTS over a season, and consider their impact on players’ playing outcomes. The results of the study revealed that the MD shows the maximum levels of training load between all the days of a competition season (i.e., MD−5, −4, and −1). Moreover, no significant difference was observed in terms of the mesocycles except for wCWL and wTS.

The main results were: (i) the MD was featured by the highest values of detected load over the week, whereas MD-1 was featured by the lowest load values over the same time frame; (ii) the highest values of wAWL, wCWL, and wTS were detected in the mid-season (e.g., W8, 9, and 13), whereas the lowest load values were detected in the early-season (e.g., W1, 2, and 7); (iii) the highest values of wACWLR were observed in the early-season (e.g., W1 and 7), whereas the lowest values were observed in the end-season (e.g., W14 and 20); and (iv) the highest values of wTM were observed in the end-season (e.g., W14 and 20), whereas the lowest values were observed in the mid-season (e.g., W8 and 13). Therefore, these results provide new insights for coaches and practitioners about perceived loads over a season in elite youth level. From a training science perspective, the TL data show that the team adopted a weekly periodization with the highest workload 3 days before a match and the lowest loads on 1 day before the match. In this context, it should be considered that the daily TL level on MD_–__3_ resulted between 350 and 400 A.U including all players in each position (Figure 1) and this could partly explain the great variability in some of the internal load variables on that day. Such TL distribution and periodization strategies for matches are in accordance with previous studies (Anderson et al., 2016; Stevens et al., 2017). The finding of a negative effect of high TL and long training duration performed 1 day before the match is not surprising. The high TL and training duration may induce fatigue (Thorpe et al., 2017), which negatively impacts performance (Rowell et al., 2018). Previous studies did not find any meaningful differences of workload metrics (e.g., AWL, CWL, ACWLR, TM, and TS) for distance-based workload between pre- and in-season blocks in professional soccer players (Los Arcos et al., 2017; Clemente et al., 2020), but this was not the case in the study of Nobari et al. (2020b).

In this study, similar findings were found for perceived load over the season, i.e., AWL 1469 A.U. (lowest values in early-season) and 1680 A.U. (highest values in mid-season), CWL 1467 A.U. (lowest values in early-season) and 1676 A.U. (highest values in mid-season), ACWLR 1.01 A.U. (lowest values in early-season) and 1.07 A.U. (highest values in end-season), TM 1.12 A.U. (lowest values in mid-season) and 1.20 A.U. (highest values in end-season), and TS 1309 A.U. (lowest values in early-season) and 1505 A.U. (highest values in mid-season). This study also found lower absolute values of AWL (1469-1680 A.U.) than in English elite U-18 (3984 ± 222 A.U.), U16 (2919 ± 136 A.U.), and U-14 age-groups (2524 ± 128 A.U.; Wrigley et al., 2012). Differently, this study provided results similar to those from another study by the same group (Nobari et al., 2020a). In all these studies, the players trained 3 days and played one match per week.

Recent literature has discussed the effects of training and matches on wellness indicators mainly in professional (Clemente et al., 2017; Costa et al., 2018), collegiate soccer players (Rabbani et al., 2018), physiological variables (Nobari et al., 2021a), and fitness status variations (Nobari et al., 2021d). In general, well-being ratings were sensitive to assessing the impact of congested fixture (Clemente et al., 2017; Rabbani et al., 2018), Ramadan fasting (Chamari et al., 2012), and match-induced fatigue (Rabbani et al., 2019), but not to the effects of late-night training with low perceived load (Costa et al., 2018). However, to the authors’ knowledge, no previous study investigated these effects in elite young players. Here, variations of training workload in micro- and meso-cycles between and within-weeks (considering playing position) were observed, indicating that the amount of training and matches was well tolerated by the players over the investigated season.

In the present study, in addition to being assessed over time (e.g., over micro- and meso-cycle), training workload was also assessed considering playing positions. Although over the micro-cycle the playing positions were very similar in terms of training workload, this was not the case over the meso-cycle. In general, there were significant differences in training workload considering playing positions between micro-cycles (e.g., early-, mid-, and end-season), whereas with within micro-cycles no significant differences were observed except in some cases (between CD and CM in early-season regarding AWL and CWL variables and between WD and WM in end-season regarding ACWLR variable). This finding provides a long-term view on training workload for soccer conditioning coaches. Also, such information has practical and theoretical value for coaches and practitioners to tailor training session distributions aiming to reach peak performance during the matches and minimizing wellness disorders.

This is one of the first studies to analyze the relationship between micro and meso cycles among elite young soccer players. Internal training load has been measured by objective variables in conjunction with a subjective scale (s-RPE) that can both be used as a replacement for objective measures but provides a cheaper and more effective means for the assessment of the loads imposed on athletes following training sessions (Bourdon et al., 2017). A reliable assessment tool for assessing the loading in a session should serve as a good indicator for coaches, as well as for practical applications in training (Akenhead and Nassis, 2016).

This study has some limitations that should be acknowledged. First, TL was only measured in terms of internal load and this may have constrained the real quantification of physiological responses to the training and matches. Second, the influence of match players’ participation in TL, original wellness status, and friendly competition between soccer players was not considered. Therefore, further studies may include distance zones, sprints, and accelerometer-based workload data to describe the weekly load variations for match starters and non-starters over the season. However, to the authors’ knowledge, this is the first study investigating the variability of internal TL considering playing position at an elite youth level. Based on that, more studies with different teams and countries should be performed to generalize the present results.

## Conclusion

This study described the weekly variations of training workload over micro- and meso-cycles considering playing position over the season in elite young soccer players. In general, the findings revealed that, in addition to MD, MD_–__3_, is also featured by a high training workload over a week. Also, the highest values of wAWL, wCWL, and wTS were found in the mid-season, whereas the lowest values were found in the early-season. The highest values of wAWL and wCWL were found in mid-season and end-season, whereas their ratio was higher in early-season. The highest values occurred for wTS in mid-season and for wTM in end-season. Reduced variability between- and within-weeks was found for perceived ratings. Finally, this research can be considered a typical outline with details of the training workload for soccer conditioning coaches.

## Data Availability Statement

The raw data supporting the conclusions of this article will be made available by the authors, without undue reservation.

## Ethics Statement

The studies involving human participants were reviewed and approved by Ethics Committee of the University of Isfahan and University of Mohaghegh Ardabili. Written informed consent to participate in this study was provided by the participants’ legal guardian/next of kin.

## Author Contributions

HN and JP-G designed the study and drafted the manuscript. HN, RV, and LPA performed the experiments. HN and RV participated in the data analysis and drafted the manuscript. HN, JP-G, and LPA critically revised the manuscript. All authors read and approved the final version of the manuscript.

## Conflict of Interest

The authors declare that the research was conducted in the absence of any commercial or financial relationships that could be construed as a potential conflict of interest.
